# Development of an age-adapted simplified acute physiology score II using first-24-hour variables in pediatric patients with congenital diaphragmatic hernia: the CDH-SAPS

**DOI:** 10.1007/s00431-026-07369-5

**Published:** 2026-09-02

**Authors:** J. Veljkovic, J. Binder, C. Krall, W. Krois, S. C. M. Cochius-den Otter, M. Hermon, J. Golej, A. Berger, Jennifer B. Brandt

**Affiliations:** 1 Division of Neonatology, Pediatric Intensive Care and Neuropediatrics, Medical University of Vienna, Waehringer Guertel 18-20, 1090, Vienna, Austria; 2 Department of Obstetrics and Feto-Maternal Medicine, Medical University of Vienna, Vienna, Austria; 3 Center for Medical Statistics, Informatics and Intelligent Systems, Medical University of Vienna, Vienna, Austria; 4 Department of Pediatric Surgery, Medical University of Vienna, Vienna, Austria; 5 Department of Neonatal and Pediatric Intensive Care, Erasmus MC Sophia Children’s Hospital, University Medical Center Rotterdam, Rotterdam, Netherlands; 6 Comprehensive Center for Pediatrics, Medical University of Vienna, Vienna, Austria

**Keywords:** Congenital diaphragmatic hernia, Risk stratification, Pediatric intensive care, Extracorporeal membrane oxygenation

## Abstract

Accurate early risk stratification in patients with congenital diaphragmatic hernia (CDH) remains challenging, as existing pediatric mortality prediction scores may not adequately reflect CDH-related pathophysiology. The main objective was to develop an early risk stratification model by adaptation of the Simplified Acute Physiology Score II (SAPS II) for mortality and extracorporeal membrane oxygenation (ECMO) requirement prediction in patients with CDH. This retrospective single-center study included pediatric patients with CDH treated at a tertiary neonatal and pediatric intensive care unit between January 2000 and December 2025. An *expanded CDH-SAPS* was developed by age adaptation and CDH-specific modification of SAPS II variables, followed by simplification for bedside applicability, resulting in *CDH-SAPS*. Both scores integrate early clinical and laboratory parameters during the first 4 h after admission complemented by 3 variables reflecting the need for major therapeutic interventions during the first 24 h. Predictive performance was compared with the Pediatric Index of Mortality 3 (PIM3) and the Score for Neonatal Acute Physiology II (SNAP-II), with the ICU mortality as primary, and ECMO requirement as secondary outcome. Among 95 included patients, the ICU mortality was 12% (*n* = 11), and 26% (*n* = 25) required ECMO support. Similar to the *expanded CDH-SAPS*, the *CDH-SAPS* demonstrated excellent discriminative ability for mortality prediction (AUROC, 0.925; 95% CI, 0.867–0.982; *p* < 0.001), with good agreement between predicted and observed outcomes. For ECMO requirement prediction *CDH-SAPS* showed moderate to good performance (AUROC, 0.789; 95% CI, 0.691–0.887; *p* < 0.001).

*Conclusion*: The *CDH-SAPS* demonstrates excellent discrimination for the ICU mortality and reliable ECMO requirement prediction in patients with CDH, supporting its use as a practical bedside tool for early clinical decision-making.

**Table:** 

**What is Known:** • *Early risk stratification in congenital diaphragmatic hernia remains challenging because general pediatric illness severity scores may inadequately reflect disease-specific risks.*
**What is New:** • *CDH-SAPS is a disease-specific, simplified bedside score that accurately predicts mortality and ECMO requirement, and supports serial assessment of disease progression.*

**What is Known:**

• *Early risk stratification in congenital diaphragmatic hernia remains challenging because general pediatric illness severity scores may inadequately reflect disease-specific risks.*

**What is New:**

• *CDH-SAPS is a disease-specific, simplified bedside score that accurately predicts mortality and ECMO requirement, and supports serial assessment of disease progression.*

## Introduction

Congenital diaphragmatic hernia (CDH) is a rare but severe congenital anomaly affecting approximately 1 in 2200–5000 live births [1]. The diaphragmatic defect allows abdominal organs to herniate into the thoracic cavity, thereby impairing lung development. This results in lung hypoplasia and often pulmonary hypertension and cardiac dysfunction, all of which are major determinants of neonatal morbidity and mortality [2]. Despite advances in intensive care, surgical techniques, and perinatal management, contributing to improved survival rates [3, 4], CDH remains a complex clinical condition requiring individualized, multidisciplinary care strategies [5]. Prognostic determinants such as the side of the defect, intrathoracic liver position, and the presence of additional congenital anomalies significantly influence outcomes [6]. As a result, accurate early risk stratification is critical for guiding therapeutic decisions, prioritizing extracorporeal membrane oxygenation (ECMO) consideration, and optimizing resource allocation in neonatal (NICU) and pediatric intensive care units (PICU) [7].

Currently available general pediatric mortality scores, including the Pediatric Index of Mortality 3 (PIM3) [8] and the Score for Neonatal Acute Physiology II (SNAP-II) [9] are not particularly designed to address the unique pathophysiological challenges of CDH [1]. SNAP-II has demonstrated predictive value for both mortality and the requirement of ECMO support in CDH, reflecting evolving standards of care [1, 9]. However, the predictive accuracy of many pediatric mortality prediction scores and models have diminished over time due to advances in care, necessitating regular recalibration [7]. This discrepancy may also account for unexpected courses in patients prenatally expected to have poor outcomes [10]. To improve outcome prediction in this vulnerable population, efforts have been made to adapt existing scores or develop disease-specific tools. Other CDH-specific prediction tools, such as the Congenital Diaphragmatic Hernia Study Group model [11] and modified versions of this model [12], have been developed but not yet been routinely implemented in daily intensive care settings. In comparison, the Simplified Acute Physiology Score II (SAPS II) is currently one of the most frequently used mortality prediction scores in adult intensive care [13, 14].

SAPS II was developed by using a large and more international patient database, which increased its reliability and generalizability. It provides a more accurate prediction of hospital mortality through an improved logistic regression model [13].

In addition to the already established pediatric scores, the aim of this study was to create an age-adapted version of the Simplified Acute Physiology Score II and modify it for use in patients with CDH.

In a two-stage scoring model development, an expanded Congenital Diaphragmatic Hernia-Simplified Acute Physiology Score (*expanded CDH-SAPS* or *eCDH-SAPS*) based on SAPS II was created and then simplified for easy use in daily clinical routine (*CDH-SAPS*).

This study aimed to evaluate the predictive and descriptive performance of the newly developed *eCDH-SAPS* and *CDH-SAPS* concerning hospital mortality and ECMO requirement in pediatric patients with CDH. The scores’ discriminatory capacity was benchmarked against the established scores PIM3 and SNAP-II to determine the clinical utility as a tailored risk stratification tool for this high-risk cohort.

## Methods

### Study design and study population

This retrospective study was conducted as a single-center investigation at the Comprehensive Center for Pediatrics of the Medical University in Vienna, and includes all pediatric patients diagnosed with left-, right, or both-sided CDH, born between January 1 st, 2000, and December 1 st, 2025. Patients born in other institutions and subsequently transferred postnatally to our center were also included.

Exclusion criteria were gestational age (GA) below 32 weeks, age older than 1 month at admission, body weight below 1500 g, and readmissions.

### Development of eCDH-SAPS and CDH-SAPS

Both scores, *eCDH-SAPS* and *CDH-SAPS*, were created in a two-stage model building process (Table 1).

**Table 1:** eCDH-SAPS and CDH-SAPS

*eCDH-SAPS*				*CDH-SAPS*	
Variables		Points	Values	Points	Values
Age [15, 16]		16	1–11 months		
		18	0–30 days		
Heart rate (per minute) [17]		11	< 104	11	< 104
		2	104–118		
		0	119–164	0	104–185
		4	165–185	7	> 185
		7	> 185		
Systolic blood pressure (mmHg) [18, 19]	0– < 1 mo	13	< 35	13	< 35
	1mo– < 1y				
	0– < 1mo	5	35–65		
	1mo– < 1y		35–75		
	0– < 1mo	0	> 65	2	> 100
	1mo– < 1y		> 75		
	0–1y	2	> 99 (Male)		
			> 100 (Female)		
Body temperature (°C) [13, 20]	Rectal	0	< 38.5	3	> 38.5 (independent of the method of measurement)
	Ear, oral, axillary, forehead		< 38.0		
	Rectal	3	> 38.5		
	Ear Oral Axillary Forehead		> 38.0		
SpO2/FiO_2_ [22–24]		11	< 148 With respiratory support	11	< 148 With respiratory support
		9	148–220 With/–out respiratory support		
		6	221–291		
		0	≥ 292		
Urinary output (ml/kg/hr) [25, 26]		11	Anuria	11	Anuria
		4	< 1.0 ml/kg/hr		
		0	> 1–2 ml/kg/hr		
BUN (mg/dL) [27, 28]		0	8–28	10	> 51
		6	29–51		
		10	> 51		
WBC (103/mm3) [27, 29]	≤ 1mo	12	< 2	12	< 2
	2–11mo				
	≤ 1mo	0	2–19.5		
	2–11mo		2–17.5		
	≤ 1mo	3	> 19.5		
	2-11mo		> 17.5		
Potassium (mmol/L) [27, 30, 31]		3	< 3.5	3	< 3.5
		0	3.5–6.0	0	3.5–6.0
		3	> 6.0	3	> 6.0
Sodium (mmol/L) [27, 32, 33]		5	< 133	5	< 133
		0	133–145	0	133–145
		1	> 145	1	> 145
SBC (mmol/L) [34, 35]	Male	6	< 15	6	< 15
	Female				
	Male	3	15–16		
	Female		15–17		
	Male	0	17–24		
	Female		18–24		
Conjugated Bilirubin (mg/dL) [36, 37]		0	< 2.0	9	> 4.9
		4	2.0–4.9		
		9	> 4.9		
GCS [13]		26	< 6	26	< 6
		13	6–8		
		7	9–11		
		5	11–13		
		0	14–15		
Reasons for admission (instead of „chronic diseases “ [8, 39–41, 44]		8	Need for iNO within the first 24 h of admission	8	Need for iNO within the first 24 h of admission
		9	GA > 32 and < 34 weeks or Birth weight > 1500 and < 2000 g*	9	GA > 32 and < 34 weeks or Birth weight > 1500 and < 2000 g*
		17	Need for CPR within the first 24 h of admission	17	Need for CPR within the first 24 h of admission
		17	Need for ECMO support within the first 24 h of admission*	17	Need for ECMO within the first 24 h of admission*
Type of admission [13, 43]		0	Scheduled surgical	8	Unscheduled
		6	Medical		
		8	Unscheduled surgical		

*The variables “need for ECMO support within the first 24 h of admission” as well as “GA between 32 and 34 weeks or birth weight between 1500 and 2000 g” were excluded for testing the outcome “requirement of ECMO support during the ICU stay”

For application in a pediatric population, the variable “age” was redefined into 5 categories: “0 to 30 days”, “1 to 11 months”, “12 to 71 months”, “72 to 143 months”, and “144 to 216 months”. Score allocation was inversely proportional to age, with the youngest group (“0 to 30 days”) receiving the highest score (18 points), reflecting the comparatively higher mortality risk observed in younger patients [15, 16].

All SAPS II variables [13] were age-adapted for use in pediatric patients under 3 months of age. Therefore, reference values for the cardiovascular parameters “heart rate” and “systolic blood pressure” were adjusted according to the age-related percentiles [17–19].

As the type of measurement plays a role in the evaluation of the variable “body temperature”, it was divided in two categories: “rectal” for central and “tympanic, oral, axillary or forehead” for peripheral body temperature measurement [13, 20, 21].

The “PaO_2_/FiO_2_ (PF) ratio” was replaced by the quotient of SpO_2_ and FiO_2_ (SF ratio) to express pulmonary impairment [22–24].

The variable “urinary output” was assessable in three categories depending on the urine production, with anuria assigned the highest number of points [25, 26].

All laboratory parameters, including “blood urea nitrogen (BUN)” [27, 28], “white blood cells (WBC)” [27, 29], “potassium” [27, 30, 31], “sodium” [27, 32, 33], “serum bicarbonate (SBC)” [34, 35], and “bilirubin” [36, 37] were adapted to age as well. The reference for “bilirubin” refers exclusively to conjugated (direct) bilirubin, because neonatal liver dysfunction is primarily associated with elevated conjugated bilirubin [36, 37].

The “Glasgow Coma Scale (GCS)” remained part of the *eCDH-SAPS*, although the pediatric version should be used when performing the GCS [13, 38]. However, the variable “type of admission” was taken directly from the original SAPS II without the need for age-adaptation [13].

The variable “chronic diseases” in the original SAPS II was replaced by “reasons for admission”, including the “need for cardiopulmonary resuscitation (CPR) within the first 24 h of admission” [8, 39], “the need for ECMO support within the first 24 h of admission” [40], “GA between 32 and 34 weeks or birth weight between 1500 and 2000 g” and the “need for inhaled nitric oxide (iNO) within the first 24 h of admission”. The highest score of 17 points was awarded for the “need for cardiopulmonary resuscitation (CPR) within the first 24 h of admission” [8, 39] and/or “the need for ECMO support within the first 24 h of admission” [40]. Since prematurity is known as a risk factor for increased mortality in patients with CDH [41], 9 points were awarded for “GA between 32 and 34 weeks or birth weight between 1500 and 2000 g”. Additionally, pulmonary hypertension is an important contributor of mortality in CDH patients, with the need for iNO therapy serving as a marker of its clinical severity [42]; hence, the “need for iNO within the first 24 h of admission” was implemented in *eCDH-SAPS* as an indicator for pulmonary hypertension.

In a second step *eCDH-SAPS* was modified to allow rapid and simplified application, resulting in the *CDH-SAPS*.

The variable “age” was excluded as the score itself was age-adapted.

The following variables were adapted by considering only the highest or lowest values for score calculation: “heart rate”, “systolic blood pressure”, “SF” ratio, “conjugated bilirubin”, “WBC”, “BUN”, “urinary output”, “body temperature”, “serum bicarbonate”, and “GCS”. The variable “type of admission” was simplified by awarding 8 points as soon as the admission was unscheduled due to unexpected events. The basis for this point distribution was the OR of 4.8 calculated by Coro et al. for PICU patients with cardiovascular diseases who were admitted unscheduled [43]. A prenatally undiagnosed CDH was equated with an unscheduled admission.

### Data management and statistical methods

Both *eCDH-SAPS* and *CDH-SAPS* were compared to PIM3 and SNAP-II to test their predictive performance.

PIM3 and SNAP-II were designed for the use on the day of admission [8, 9]. Data from the first hours after admission to NICU or PICU were considered for score calculation (Table 2).

**Table 2:** Comparison of PIM3 and SNAP-II

	PIM3		SNAP-II
Variables	pupils fixed to light (Yes/No)		MBP 20–29 mmHg
	elective admission (Yes/No)		MBP < 20 mmHg
	mechanical ventilation in the first hour (Yes/No)		lowest temperature 95°–96° F
	absolute value of base excess (mmol/L)		PO_2_/FiO_2_ ratio 1.0–2.49
	SBP at admission (mmHg)		PO_2_/FiO_2_ ratio 0.3–0.99
	SBP^2^/1000		PO_2_/FiO_2_ ratio < 0.3
	100 × FiO_2_/PaO_2_ (mmHg)		lowest serum pH 7.10–7.19
	Recovery from	cardiac bypass procedure	lowest serum pH < 7.10
		cardiac non–bypass procedure	multiple seizures
		other, non-cardiac procedure	urine output 0.1–0.9 ml/kg/h
	Very high-risk diagnosis		urine output < 0.1 ml/kg/h
	high-risk diagnosis		
	low-risk diagnosis		
Data collection window	first hour after admission		first 12 h after admission
Outcome	death in ICU		in-hospital mortality

*SBP*  systolic blood pressure, *FiO*_*2*_  fraction of inspired oxygen, *PaO*_*2*_ arterial oxygen partial pressure, *MBP*  mean blood pressure; °C = (°F-32)/1.8

The newly developed *eCDH-SAPS* and *CDH-SAPS* incorporate initial clinical and laboratory abnormalities recorded within the first 4 h after NICU or PICU admission, together with 3 variables reflecting the need for major therapeutic interventions (starting iNO, CPR, and the need for ECMO, and) during the first 24 h. Both assessment windows were defined relative to the time of admission to the NICU or PICU, irrespective of the infant’s place of birth.

Additionally*, eCDH-SAPS* and *CDH-SAPS* were re-evaluated after 24, 48, and 72 h after admission to evaluate the change over time. The 4-h time window was always adhered to.

If a variable had several values recorded during the specific time window, the worst recorded value was used for scoring, reflecting the patients’ most critical clinical state. Missing data, primarily laboratory parameters, were unavailable when not part of routine monitoring or when measurement was deemed clinically unnecessary.

“Mortality during the intensive care unit (ICU) stay” was defined as the primary outcome and the “requirement of ECMO support during the ICU stay” as the secondary outcome. To predict the outcome “requirement of ECMO support during the ICU stay”, the variable “need for ECMO support within the first 24 h of admission” was excluded and only parameters assessed prior to the start of ECMO were considered for risk calculation. Since ECMO is not recommended in patients with GA below 34 weeks or birth weight below 2000 g [44], the variable “GA between 32 and 34 weeks or birth weight between 1500 and 2000 g” was also excluded for testing the secondary outcome.

Descriptive statistics were applied to characterize the study population, including median, interquartile range (IQR), mean, and standard deviation for continuous variables, frequencies, and percentages for categorical variables.

The relationship between each score and both outcomes was assessed using binary logistic regression and chi-squared tests.

To evaluate the predictive performance of each scoring system, including their discriminative ability between survivors and non-survivors and their accuracy of predicting observed outcomes, the area under the receiver operating characteristic curve (AUROC) and Hosmer–Lemeshow goodness-of-fit (GOF) tests were applied. Statistical significance of AUROC differences was tested using the “ROC Analysis” function in IBM SPSS statistics (Version 28.0.0.0, IBM corporation, USA). Score progression was analyzed using the Friedman test and post hoc methods.

## Results

During the study period a total of 106 patients with CDH were admitted to our NICU and PICU of whom 12 patients died (11%) during their hospital stay.

For the analysis, 95 patients were included, with an ICU mortality rate of 12% (*n* = 11). Eleven patients were excluded. One of these patients died immediately after birth, and six patients were excluded because of missing data attributable to incomplete documentation of parameters in paper-based medical records from the early 2000s. Four patients did not meet the inclusion criteria.

A total of 30 patients (32%) were born preterm (GA between 32 + 0 and 36 + 6), and in 26 patients (27%) CDH had not been diagnosed prenatally (unscheduled admission). A detailed description of demographic data is presented in Table 3.

**Table 3:** Demographic data

Gender; *N* (%)	
Female	35 (27)
GA at admission	
Median (weeks)	38 + 0, IQR: 36 + 2–39 + 0
Birth weight	
Median (kg)	3.0, IQR: 2.6–3.3
Missing data; N (%)	1 (1)
Birth mode; *N* (%)	
Caesarean section	75 (79)
Spontaneous delivery	15 (16)
Missing data	5 (5)
Defect side; *N* (%)	
Left	80 (84)
Right	14 (15)
Both	1 (1)
Liver position; *N* (%)	
Intrathoracic	50 (53)
Abdominal	41 (43)
Missing data	4 (4)
Operation method; *N* (%)	
Stitch	54 (57)
Patch	39 (41)
No repair	2 (2)

Beyond CDH, 79 patients (83%) presented with additional comorbidities or complications. The most common comorbidity was pulmonary hypertension (45%), followed by other cardiovascular (37%), and respiratory disorders (32%).

The mean duration of mechanical ventilation was 17.0 days (95% CI: 13.5–21.4; SD = 20.1). Inhaled NO or sildenafil was administered in 69% of patients. Additionally, 14% received ECMO support within the first 24 h of admission due to clinical deterioration, and 11% of patients required CPR. In total, CDH was surgically repaired in 93 patients. In two patients, repair was not possible because of death despite ECMO support.

### Primary outcome: mortality during the ICU stay

Both scores, *eCDH-SAPS* and *CDH*-*SAPS* were assessed at multiple time points to evaluate their discriminative performance. Overall, *eCDH-SAPS* and *CDH-SAPS* demonstrated good agreement between the predicted and observed mortality (Table 4).

**Table 4:** AUROC and goodness-of-fit (GOF) for the outcome “mortality”

Timepoint	AUROC (95% CI) *p* value		AUROC difference, *p* value	GOF *p* value	
	*eCDH-SAPS*	*CDH-SAPS*		*eCDH-SAPS*	*CDH-SAPS*
Within first 4 h of admission	0.915 (0.846–0.983) *p* < 0.001	0.925 (0.846–0.983) *p* < 0.001	0.01 *p* = 0.50	*χ*^2^ = 1.6 df = 7 *p* = 0.98	*χ*^2^ = 5.5 df = 7 *p* = 0.60
12 h after admission	0.892 (0.812–0.973) *p* < 0.001	0.893 (0.814–0.972) *p* < 0.001	0.001 *p* = 0.98	*χ*^2^ = 7.9 df = 8 *p* = 0.44	*χ*^2^ = 3.0 df = 6 *p* = 0.81
24 h after admission	0.858 (0.730–0.986) *p* < 0.001	0.901 (0.813–0.989) *p* < 0.001	0.043 *p* = 0.11	*χ*^2^ = 4.0 df = 7 *p* = 0.78	*χ*^2^ = 11.7 df = 8 *p* = 0.17
36 h after admission	0.907 (0.805–1.009) *p* < 0.001	0.920 (0.825–1.015) *p* < 0.001	0.012 *p* = 0.34	*χ*^2^ = 8.3 df = 7 *p* = 0.30	*χ*^2^ = 9.5 df = 7 *p* = 0.22
72 h after admission	0.878 (0.757–1.000) *p* < 0.001	0.862 (0.733–0.991) *p* < 0.001	0.017 *p* = 0.21	*χ*^2^ = 5.0 df = 8, *p* = 0.76	*χ*^2^ = 2.6 df = 6 *p* = 0.85

*AUROC*  area under the receiver operating characteristic curve, *GOF * goodness-of-fit (Hosmer–Lemeshow test), *CDH-SAPS * Congenital Diaphragmatic Hernia – Simplified Acute Physiology Score, *eCDH-SAPS*  expanded CDH-SAPS, *df*  degrees of freedom

The highest AUROC for mortality prediction was observed for *CDH*-*SAPS* (AUROC = 0.925, *p* < 0.001, 95% CI: 0.867–0.982), followed by *eCDH-SAPS* (AUROC = 0.915, *p* < 0.001, 95% CI: 0.846–0.983), PIM3 (AUROC = 0.769, *p* = 0.001, 95% CI: 0.605–0.933) and SNAP-II (AUROC = 0.664, *p* = 0.11, 95% CI: 0.451–0.877). There was a significant difference in AUROC between *eCDH-SAPS* and both PIM3 and SNAP-II (*p* = 0.04 and *p* = 0.01, respectively), and between *CDH*-*SAPS* and both PIM3 and SNAP-II (*p* = 0.03 and *p* = 0.01, respectively), in favor of *eCDH-SAPS* and *CDH*-*SAPS*. In contrast, the difference between *eCDH-SAPS* and *CDH*-*SAPS* was not statistically significant (*p* = 0.50), nor was the difference between or PIM3 and SNAP-II (*p* = 0.34). All scores showed acceptable calibration, with non-significant GOF-test results (*p* > 0.05).

**Fig. 1:**
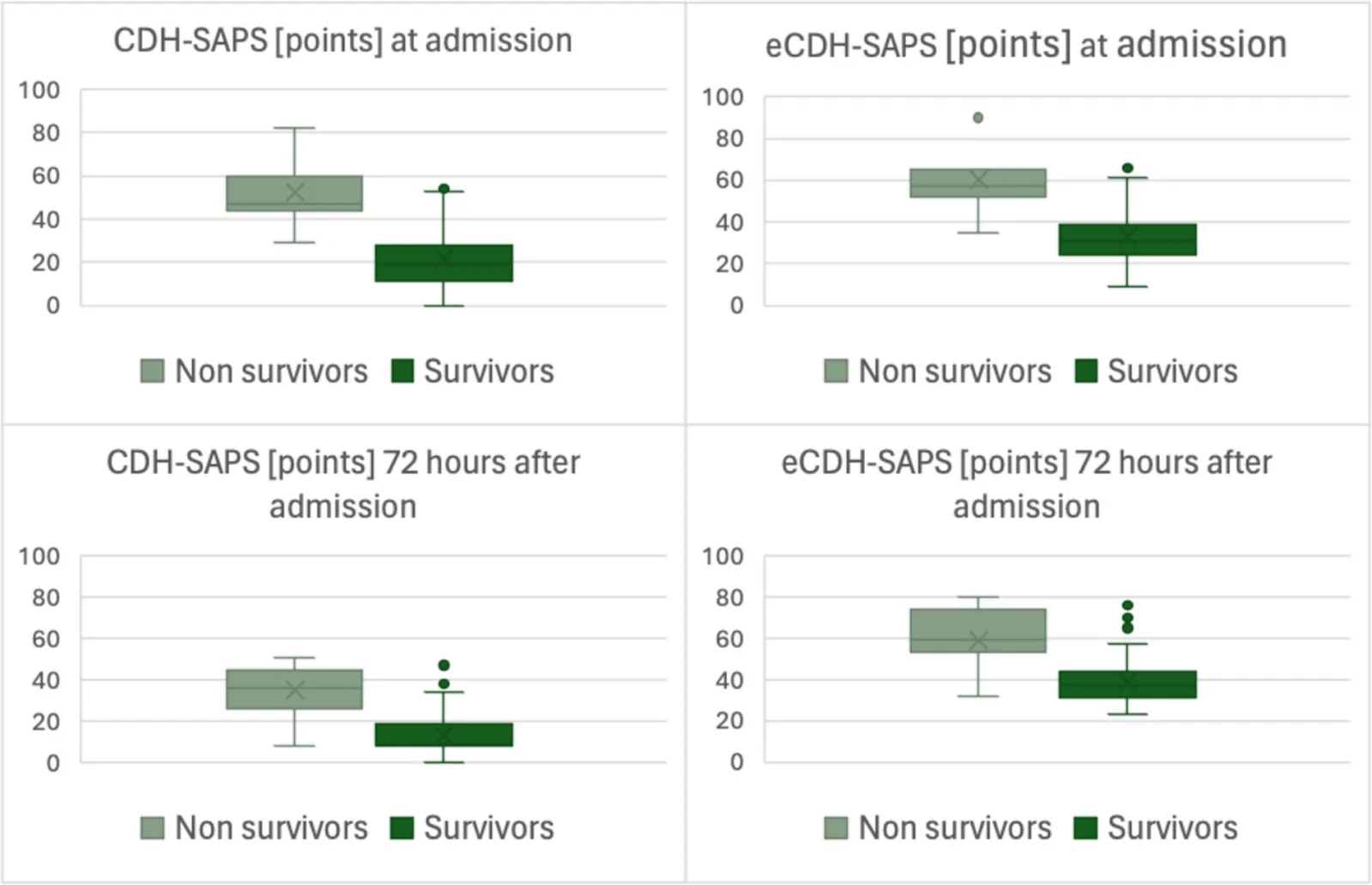
Survivors compared to non-survivors at admission and 72 h after admission

Furthermore, there was no overlap between the 75th percentile of survivors and the 25th percentile of non-survivors, indicating robust separation (Fig. 1).

In survivors, the mean *eCDH-SAPS* score was 31.7 at admission (95% CI: 29.0–34.5; SD = 13.4) and 38.0 at 72 h of life (95% CI: 35.4–40.6; SD = 11.9). In comparison, survivors had an average *CDH*-*SAPS* of 21.3 at admission and of 12.2 at 72 h of life (95% CI: 10.0–14.6; SD = 10.9).

In non-survivors, the mean score at admission was 60.4 for *eCDH-SAPS* (95% CI: 51.6–71.2; SD = 17.6) and 52.5 for *CDH*-*SAPS* (95% CI: 44.5–61.7; SD = 15.6), decreasing to 57.3 after 72 h for *eCDH-SAPS* (95% CI: 50.8–67.3; SD = 14.6) and 34.7 for *CDH*-*SAPS* (95%CI: 26.5–42.2; SD = 14.0).

There was a significant score progression in survivors for eCDH-SAPS (*X*^2^ = 91.3, *p* < 0.001, df = 4) and *CDH*-*SAPS* (X^2^ = 69.5, *p* < 0.001, df = 4). Similar score progression was also found in non-survivors for *CDH*-*SAPS* (*X*^2^ = 29.1, *p* < 0.001, df = 4), but not for *eCDH-SAPS* (*X*^2^ = 5.4, *p* = 0.25, df = 4).

There was a strong association between the variable “reasons for admission” and the primary outcome “mortality” during the ICU stay: *χ*^2^ = 38.83, *p* < 0.001.

### Secondary outcome: requirement of ECMO support during the ICU stay

In total, 25 patients required ECMO support. ECMO was initiated within the first 24 h of admission in 52% of these patients (*n* = 13) and in 88% within 48 h (*n* = 22). The *eCDH-SAPS* achieved an AUROC of 0.790 (95% CI: 0.698–0.882, *p* < 0.001), and *CDH*-*SAPS* achieved an AUROC of 0.789 (95% CI: 0.691–0.887, *p* < 0.001), indicating moderate to good discriminative ability for both scores. The GOF confirmed acceptable model calibration for *eCDH-SAPS* (*X*^2^ = 9.7, df = 7, *p* = 0.2). The average score for patients not requiring ECMO was 47.5 for *eCDH-SAPS* (95% CI: 44.7–50.5, SD = 12.1) and 19.1 for *CDH-SAPS* (95% CI: 16.0–22.4, SD = 13.7). In comparison, patients who required ECMO support had a mean *eCDH-SAPS* of 59.6 (95% CI: 55.2–65.3, SD = 12.8) and a mean *CDH*-*SAPS* of 32.2 (95% CI: 27.4–38.0, SD = 13.6).

The “requirement of ECMO support” showed a significant dependence on the “need for CPR within the first 24 h of admission” and/or “need for iNO within the first 24 h of admission” (*χ*^2^ = 28.2, *p* < 0.001).

**Fig. 2:**
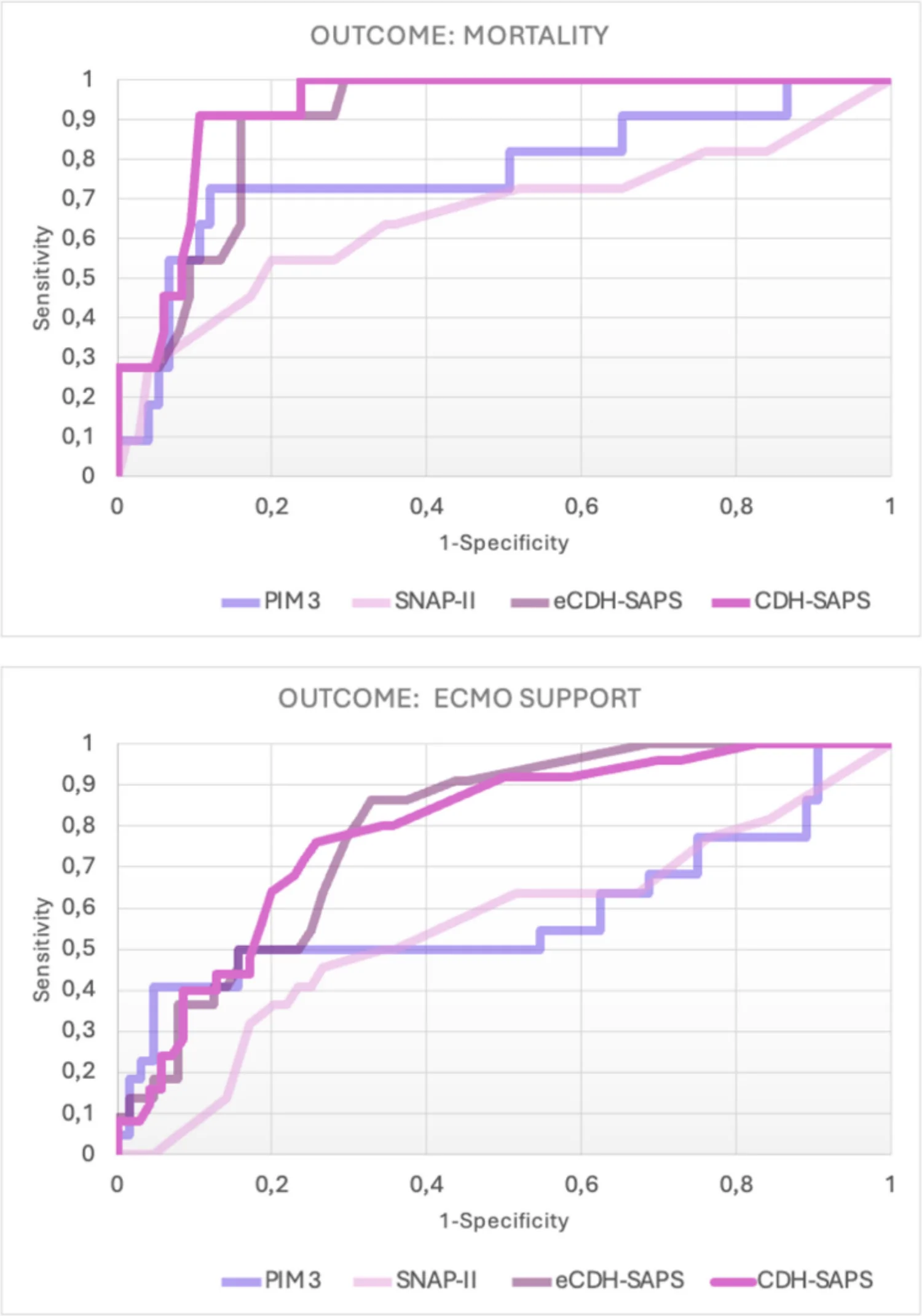
AUROC comparison for both outcomes

Since both PIM3 and SNAP-II are admission-based scores, comparisons for AUROC calculation were limited to data from the first 4 h after admission. The AUROC values and the GOF results were used to compare the scores in predicting both mortality and requirement of ECMO support (Fig. 2).

Compared to PIM3 (AUROC = 0.617, *p* = 0.14, 95% CI: 0.463–0.770) and SNAP-II (AUROC = 0.536, *p* = 0.61, 95% CI: 0.399–0.672), *eCDH-SAPS* (AUROC = 0.790, *p* < 0.001, 95% CI: 0.698–0.882) and *CDH*-*SAPS* (AUROC = 0.789, *p* < 0.001, 95% CI: 0.691–0.887) achieved the highest AUROC values. Statistically significant differences in AUROC were observed between *eCDH-SAPS* and both PIM3 and SNAP-II (*p* = 0.02 and *p* < 0.001, respectively), as well as between *CDH*-*SAPS* and both PIM3 and SNAP-II (*p* = 0.01 and *p* < 0.001, respectively). No significant AUROC difference was found between *eCDH-SAPS* and *CDH*-*SAPS* (*p* = 0.97) or between PIM3 and SNAP-II (*p* = 0.25). Good calibration for ECMO prediction was demonstrated by *CDH*-*SAPS*, PIM3 and SNAP-II, with non-significant GOF test results (*p* > 0.05). In comparison, *eCDH-SAPS* showed a significant difference between observed and expected need for ECMO support (*p* = 0.02), indicating poor calibration.

## Discussion

In the postnatal care for pediatric patients with CDH, early stratification of mortality risk and the anticipated requirement of ECMO support is critical. However, the rarity of CDH has limited the development and robust validation of disease-specific prediction models. Although the CDH severity prediction rule [11, 12] yielded promising results, it was developed and validated in relatively small cohorts. Conversely, general mortality scores were derived from larger populations but may not adequately capture the distinct pathophysiology of CDH. PIM3 has demonstrated strong performance in heterogeneous pediatric intensive care cohorts [8, 45], yet remains unvalidated specifically in CDH. By contrast, SNAP-II has been evaluated in several CDH cohorts and has shown good predictive performance [9, 10, 46–48].

Although SAPS II was originally developed for adult patients and is therefore not intended for use in pediatric patients, its development based on a large international patient dataset, its worldwide validation and application provide a strong methodical foundation [13]. Building on this established mortality prediction framework, we developed an age-adapted CDH-specific modification of SAPS II (*eCDH-SAPS*) and simplified it for easier use in patients with CDH (*CDH-SAPS*). Both scores combine early clinical and laboratory abnormalities recorded within the first 4 h of admission with indicators of major therapeutic escalation, as iNO, CPR, and ECMO during the subsequent 24-h period. Rather than serving as isolated predictors, these interventions contextualize the initial physiological derangements and capture the early trajectory of disease severity, thereby enabling a more comprehensive assessment of mortality risk.

In this study, *eCDH-SAPS* and *CDH*-*SAPS* demonstrated strong discriminative performance in predicting mortality during the ICU stay, with AUROC values of both scores > 0.9, indicating excellent discriminatory ability [20]. Furthermore, *eCDH-SAPS* and *CDH-SAPS* showed appropriate calibration, as no significant differences were shown between observed and expected mortality. The lowest mortality rate was found in patients with scores between 0 and 30 points for both *eCDH-SAPS* and *CDH-SAPS*. This finding is broadly consistent with the original SAPS II, which formed the basis for *eCDH-SAPS* and *CDH*-*SAPS *[49].

Beyond the meaningful mortality risk stratification performance, *eCDH-SAPS* and *CDH-SAPS* differed in their ability to capture changes in clinical condition over time. *CDH-SAPS* showed significant temporal progression in both survivors and non-survivors, whereas *eCDH-SAPS* demonstrated a comparable trajectory only among survivors. This dynamic behavior and reproducibility suggest that *CDH-SAPS* may be particularly suitable for serial assessment. Such an approach is clinically relevant in CDH, where an initial period of relative stability can be followed by rapid clinical deterioration [50], and serial assessment may therefore facilitate the detection of subtle changes in disease severity and support timely reassessment of mortality risk.

Compared to the mortality risk prediction, a similar graded association was observed for ECMO requirement. In both scores, *eCDH-SAPS* and *CDH-SAPS*, the predicted probability of ECMO support was approximately 10% or less at scores of up to 20 points and increased to approximately 10–15% at higher score ranges of up to 30 points. These findings suggest that increasing score values are associated with a higher likelihood of requiring ECMO support. However, calibration differed between *eCDH-SAPS* and *CDH-SAPS*. The GOF analysis indicated adequate agreement between observed and predicted ECMO requirement for *CDH-SAPS,* but not for *eCDH-SAPS*. These findings suggest that CDH-SAPS provides more reliable estimates and is therefore the preferable score for prediction of ECMO requirement.

To further evaluate the performance of the newly developed scores, *eCDH-SAPS* and *CDH-SAPS* were compared with the established pediatric mortality scores, PIM3 [8] and SNAP-II [9]. The validated prediction rule for the severity of CDH [12] was not included in this comparison, because one of its variables, “low birth weight, < 1500 g”, constituted an exclusion criterion in our study, as it is an exclusion criteria for ECMO support. Compared with SNAP-II [9] and PIM3 [8], *eCDH-SAPS* and *CDH*-*SAPS* showed superior discriminative performance in predicting both “mortality” and “requirement of ECMO support” during the ICU stay. This comparison supports the potential clinical relevance of *eCDH-SAPS* and, especially, *CDH-SAPS* as disease-specific tools for early risk stratification in this high-risk population.

### Limitations

The main limitation of this study is the small sample size and the presence of missing data inherent to the retrospective data analysis. The small sample size was due to the rarity of CDH, necessitating a long retrospective observation period because of the single-center design.

While external validation is required for broader implementation of the *CDH-SAPS,* differences in patient populations and management strategies across centers should be considered when interpreting validation results.

Also, the long inclusion time has limitations. During the almost 26-year study period, there were changes in ventilation strategies, ECMO indications, surgical timing, and pulmonary hypertension therapy, as also described in a multicenter registry study, including more than 5.000 CDH patients over comparable time period [51].

In this study cohort, the admission day was mostly the first day of life. Immediately after birth, some patients were in life-threatening conditions, with clinical efforts focused primarily on restoring or maintaining cardiorespiratory stability. As a result, the determination of certain variables, such as WBC, BUN or bilirubin, was not prioritized and often performed at a later stage, mostly outside the observed time window. Additionally, in cases of resuscitation, the measurement of certain variables, such as systolic blood pressure, was often not feasible. Both scenarios contributed to missing data.

The GCS was not available for any patients in our study, as all patients were sedated, intubated, and mechanically ventilated within the first few hours of life. Nevertheless, we have retained the variable as part of the score, as we did not want to alter the basic structure of the score.

## Conclusion

Based on SAPS II, the *eCDH-SAPS* and the simplified *CDH-SAPS* were developed for mortality prediction in patients with CDH. The simplified *CDH-SAPS* retained the mortality prediction performance of the more complex *eCDH-SAPS* while improving bedside applicability using fewer variables. Furthermore, *CDH-SAPS* demonstrated superior performance in predicting ECMO requirement and capturing clinical progression over time. These results support the potential utility of *CDH-SAPS* as a simple and clinically applicable risk assessment tool for patients with CDH.

To facilitate the application and external validation of the CDH-SAPS, we developed the CDH-SAPS calculator. The calculator is freely available for clinical and research use at the following link: https://n1k0la-21.github.io/CDH-SAPS/. Password: mWhld83kDu231nZaQ.

## Data Availability

No datasets were generated or analysed during the current study.

## Abbreviations

*AUROC*: Area under the receiver operating characteristic curve
*CDH*: Congenital diaphragmatic hernia
*CI *: Confidence interval
*CPR*: Cardiopulmonary resuscitation
*ECMO*: Extracorporeal membrane oxygenation
*FiO*_2_: Fraction of inspired oxygen
*GA*: Gestational age
*GOF*: Goodness of fit
*IQR*: Interquartile range
*iNO *: Inhaled Nitric Oxide
*NICU*: Neonatal intensive care unit
*PICU*: Pediatric intensive care unit
*PIM3*: Pediatric Index of Mortality 3
*PaO*_2_: Arterial oxygen partial pressure
*SAPS*: Simplified Acute Physiology Score
*SpO*_2_: Peripheral oxygen saturation
*SNAP-II*: Score for Neonatal Acute Physiology II
